# Synthesis and application of cationised cellulose for removal of Cr(VI) from acid mine-drainage contaminated water

**DOI:** 10.12688/aasopenres.13182.1

**Published:** 2021-01-21

**Authors:** Anita Etale, Dineo S. Nhlane, Alseno K. Mosai, Jessica Mhlongo, Aaliyah Khan, Karl Rumbold, Yannick B. Nuapia

**Affiliations:** 1 Global Change Institute, University of the Witwatersrand, Johannesburg 2000, South Africa, Johannesburg, Gauteng, 2000, South Africa; 2 Molecular Sciences Institute, School of Chemistry, University of the Witwatersrand, Johannesburg 2000, South Africa, Johannesburg, Gauteng, 2000, South Africa; 3 School of Molecular and Cell Biology, University of the Witwatersrand, Johannesburg 2000, South Africa, Johannesburg, Gauteng, 2000, South Africa

**Keywords:** Cellulose cationisation, Chromium(VI), acid mine drainage, hemp cellulose, glycidyltrimethylammonium chloride, 3-chloro-2-hydroxypropyl trimethyl ammonium chloride, quaternary ammonium salts

## Abstract

**Background: **Acid mine drainage (AMD) leads to contamination of surface and ground water by high levels of toxic metals including chromium. In many cases, these waters are sources of drinking water for communities, and treatment is therefore required before consumption to prevent negative health effects.

**Methods:** Cationised hemp cellulose was prepared by etherification with two quaternary ammonium salts: 3-chloro-2-hydroxypropyl trimethyl ammonium chloride (CHPTAC) and glycidyltrimethylammonium chloride (GTMAC) and examined for (i) the efficiency of Cr(VI) removal under acid mine-drainage (AMD) conditions, and (ii) antibacterial activity. Adsorbents were characterised by electron microscopy, Fourier transform infrared (FTIR), CP-MAS ^13^C nuclear magnetic resonance (NMR) spectroscopy, elemental composition and surface charge.

**Results:** FTIR and solid state ^13^C NMR confirmed the introduction of quaternary ammonium moieties on cellulose. ^13^C NMR also showed that cationisation decreased the degree of crystallisation and lateral dimensions of cellulose fibrils. Nevertheless, 47 %  - 72 % of Cr(VI) ions were removed from solutions at pH 4, by 0.1 g of CHPTAC and GTMAC-cationised cellulose, respectively. Adsorption kinetics followed the pseudo-second order model and isotherms were best described by the Freundlich and Dubinin-Radushkevich models. When GTMAC-modified cellulose was applied to AMD contaminated water (pH 2.7); however, Cr(VI) removal decreased to 22% likely due to competition from Al and Fe ions. Cationised materials displayed considerable antibacterial effects, reducing the viability of *Escherichia coli* by up to 45 % after just 3 hours of exposure.

**Conclusions:** Together, these results suggest that cationised cellulose can be applied in the treatment of Cr(VI)-contaminated mine water particularly if pre-treatments to reduce Fe and Al concentrations are applied.

## Introduction

Acid mine drainage represents a major source of water contamination in regions of previous mining activity ( McCarthy, 2010; Naicker *et al.,* 2003). A number of processes are responsible for this contamination, most notably the weathering of waste piles and leaching of toxic ions through these piles into groundwater, or surface run-off of to surface water bodies ( Tutu, 2006). Concentrations as high as 152 mg kg ^-1^ of chromium have been reported in fly ash ( Saha *et al.,* 2011), which is thought to be responsible for the elevated chromium levels of soils around coal-fired power plants. Although it exists as the relatively immobile Cr(III) under reducing conditions, oxidising conditions are characterized by highly mobile Cr(VI) which is classified as a carcinogen. As such, the WHO limits for chromium in drinking water are set at 50 µg L ^-1 ^( WHO, 2003).

A number of approaches exist for the removal of Cr(VI) from water, including oxidation-reduction reactions with iron oxides ( Johnston & Chrysochoou, 2012; Liu *et al.,* 2018), electrocoagulation ( Martín-Domínguez *et al.,* 2018), ion exchange ( Kabengi *et al.,* 2017). Reverse osmosis and nanofiltration membranes have also been explored for Cr removal from drinking water ( Anand *et al.,* 2018). However, as is common with membrane technologies, fouling, and high investment and operational costs present a significant barrier to wide-spread adoption.

Adsorption-based approaches are attractive for a wide range of reasons including cost-effectiveness, reusability, the possibility to apply a wide range of adsorbents, and to increase their efficiency and specificity by functionalisation with appropriate moieties ( Benhamou *et al.,* 2014; Dindar *et al.,* 2015; Etale *et al.,* 2020b; Etale *et al.,* 2015; Etale *et al.,* 2014; Nhlane *et al.,* 2020; Zou *et al.,* 2011). A wide range of adsorbents including iron oxides, porous silica, and zeolites have been shown to be effective for the treatment of water contaminated by chromium ( Benhamou *et al.,* 2013; Fellenz *et al.,* 2017; Liu *et al.,* 2018; Pour *et al.,* 2015; Sannino *et al.,* 2009; Smith & Ghiassi, 2006). In recent years, there has been shifting away from simply providing solutions, to doing so in ways that are cleaner, greener, and more affordable. Besides ensuring that solutions do not create new problems, e.g. new classes of environmental pollutants, this approach can also lead to exploitation of locally available resources, thus generating affordable solutions.

Cellulose is the most abundant natural polymer, given that it makes up a considerable fraction of the structure of plants ( Foster *et al.,* 2018). With an annual production of over 7 billion kilograms ( Garside, 2020), cellulose is considered an inexhaustible source of environmentally-friendly raw materials ( Klemm *et al.,* 2005). For the production of low-cost products, however, it is important that locally available materials, including waste materials are explored as these are often abundant and lower in cost. The extraction of cellulose from agricultural wastes including sugarcane bagasse, wheat and rice straw, jute and hemp fibres, as well as pineapple and palm leaves has been reported ( Cherian *et al.,* 2010; El Achaby *et al.,* 2018; Kassab *et al.,* 2020a; Kassab *et al.,* 2020b; Kassab *et al.,* 2019; Mandal & Chakrabarty, 2011; Rashid & Dutta, 2020; Silvério *et al.,* 2013). The application of cellulose from these waste materials for water treatment is of increasing scientific interest due to the potential affordability of adsorbent materials. Once extracted, cellulose fibres can be oxidized to imbue them with negative surfaces suitable for cation adsorption ( Beck *et al.,* 2015; Besbes *et al.,* 2011; Eyley & Thielemans, 2014; Gorgieva *et al.,* 2019; Zhao *et al.,* 2017), or cationised to allow for the adsorption of anions ( Hashem & El-Shishtawy, 2001; Udoetok *et al.,* 2016a; Udoetok *et al.,* 2016b).

A common cationization approach involves the use of quaternary ammonium salts. Traditionally performed to improve dye retention of fibres ( Acharya *et al.,* 2014; Alebeid & Zhao, 2016), cationization has also been used in liquid-liquid extraction of chromium from water ( Wang *et al.,* 2011), and to improve the performance of adsorbents for water treatment. Peat modified with glycidyltrimethylammonium chloride was shown to increase removal of phosphate and sulphate ions due to the increase in positive surface charges on the adsorbent from protonated amines ( Gogoi *et al.,* 2019; Sehaqui *et al.,* 2016). Cationization was also reported to improve the dispersion and stability of cellulose fibres in water ( Zaman *et al.,* 2012). For water treatment, cationised adsorbents proffer additional benefits due to their antibacterial properties. Polyvinyl films with GTMAC were found to be active against both Gram-positive ( *Staphylococcus aureus* and *Bacillus subtilis*) and Gram-negative ( *Escherichia coli* and *Pseudomonas aeruginosa*) bacteria ( Pour *et al.,* 2015). Cationization can also increase antibacterial activity by providing anchorage for other antibacterial agents e.g. silver nanoparticles ( Shateri Khalil-Abad *et al.,* 2009).

We investigated the cationization of cellulose fibres derived from hemp plants. Hemp represents an increasingly available feedstock for cellulose production in South Africa due to recent legalization of its cultivation for medicinal use. After extraction of oil from seeds, other plant parts including stems and branches are unused and provide a valuable source of cellulose. Cellulose was extracted via alkali treatment and cationised using two quaternary ammonium salts: 3-chloro-2-hydroxypropyl trimethyl ammonium chloride, and glycidyltrimethylammonium chloride. The efficiency of adsorbents for the removal of Cr(VI) ions was investigated at pH 4 in order to establish adsorbent efficiencies under mine drainage conditions. As surface water is often also contaminated by biological agents, the antibacterial activity of the adsorbents was quantified.

## Methods

### Materials

All chemicals used were of analytical grade. 3-chloro-2-hydroxypropyl trimethylammonium chloride (CHPTAC; 60 wt% in H _2_O, Mw = 188.10), and glycidyltrimethylammonium chloride (GTMAC; > 90 wt % in H _2_O, Mw = 151.63), dipotassium chromate (K _2_CrO _4_), L-glutathione reduced (Mw = 307.32), 5,5′-Dithiobis(2-nitrobenzoic acid) (Ellman’s reagent), Tris-HCl buffer, Na _2_HPO _4_.7H _2_O, NaH _2_PO _4_H _2_O, and NaCl were from Sigma Aldrich (South Africa). Tetrahydrofuran (THF, 99.5 % purity) was from Merck (Germany). Hydrochloric acid (HCl, 32 %), nitric acid (HNO _3_, 55 %, sodium hydroxide (NaOH), sodium chlorite (NaClO _2_), glacial acetic acid (CH _3_COOH) were from Ace Chemicals (South Africa). Dry hemp branches from which fibres were obtained were sourced locally. All solutions were prepared with double deionised water with a resistivity of 18.2 MΩ cm ^-1^. Glassware and polypropylene vials were washed, soaked in a 1M HNO _3_ acid bath for at least 24 hours, and rinsed with deionised water before use.

### Extraction of cellulose fibres

Cellulose fibres were extracted from dried stems and branches of *Cannabis sativa* plants. The stems and branches were soaked in water overnight and the fibres peeled off the woody cores. They were then subjected to alkali treatment using 4 wt % NaOH at 80 °C in three treatment rounds, each lasting 2 hours. The cellulose microfibers obtained were bleached using equal parts of NaClO _2 _(1.7 wt %) and acetate buffer (pH 4.8) prepared from 27 g NaOH and 75 mL glacial acetic acid, diluted to 1L. Three, hour long, bleaching rounds were performed. The ratio of fibre to liquor was maintained at 1:20 for each bleaching round. The extracted cellulose was then washed with deionised water several times and dried at ambient temperature.

### Cationization of cellulose adsorbents

Dried cellulose was cationised according to the procedure described by Odabas and colleagues ( Odabas *et al.,* 2016). Briefly, 5 g of cellulose fibres were added to 225 mL THF in a two-necked round-bottom flask equipped with a condenser, and stirred for 1 hour at ambient temperature (21 ± 2°C). NaOH (2.5 mL, 10 M) was then added and the mixture stirred for 30 minutes, after which 17.8 mL of CHPTAC or GTMAC was added dropwise, and the etherification reaction allowed to proceed for 15 hours at 40°C. Afterwards, the reaction was stopped by the addition of 4 M HCl (12.5 mL). The cationization solution was decanted, the fibres washed severally in ultrapure water until the pH of wash solutions was stable. Fibres were then dried in the oven at 45°C prior to characterisation and use in experiments. 

### Characterisation of cationised cellulose adsorbents

The morphology of cationised fibres was evaluated by field-emission scanning electron microscopy (FESEM). For this, the fibres were mounted on a stub with double-sided tape and sputter-coated with gold and palladium before being examined using a Zeiss Sigma 300 FESEM (Jena, Germany). The chemical structure of cellulose, before and after cationization was determined by FTIR and ^13^C NMR spectroscopy. FTIR spectra were recorded in the range 4000–500 cm ^-1^ in the absorption mode using a Tensor 27 Infrared Spectrometer (Massachusetts, USA).

Solid-state NMR experiments were performed with a Bruker Avance III spectrometer (500 MHz Bruker-BioSpin, GmbH, Rheinstetten, Germany) with a 4mm solids probe. The ^13^C MAS Spectra were recorded at 125.77 MHz at room temperature with a contact time for cross-polarization at 2 ms and a recycle delay of 5 s. The chemical shifts were calibrated using adamantane as an external chemical shift reference. Spectra were recorded after scans ranging from 1688 (pure cellulose), to 3,810 (GT-cellulose) and 4125 (CH-cellulose). Peak deconvolution was performed using MestReNova software (version 0.0.1). This analysis could also be performed using Bruker Topspin or ACD Labs software.

The pH _PZC_ of cellulose and derivatives was determined by salt titration method ( Udoetok *et al.,* 2016a). For this, a 0.01M NaCl solution was prepared and 25 mL aliquots transferred to five polypropylene tubes. The pH of these solutions was then adjusted between 2 and 10 using 0.1 M NaOH and 0.1 M HNO _3_ so that each tube had a different pH value. Cellulose and cationised derivatives (0.1 g) were then added to the NaCl solutions and suspensions equilibrated on a horizontal shaker for 48 hours. The final pH of each solution was plotted against the initial pH of the solutions and the pH _PZC_ for each material determined from intersection points.

The elemental composition of the cationised cellulose was determined at a temperature >1000°C using a Vario EL Cube elemental analyser (Elementar Analysesysteme, Langenselbold, Germany).

### Degree of Substitution (DS)

The degree of substitution was calculated from the nitrogen content as determined by elemental analysis using the Equation 1 ( Zaman *et al.,* 2012):

$$DS=\frac{162\;\times N\%}{1400-(Mw\;\times N\%)}\;(1)$$

Where, 162 is the molecular weight of anhydroglucose unit, N % is the proportion of nitrogen in CH-cellulose or GT-cellulose, and *Mw* is the molecular weight of the cationisation agent (151.63 and 188.10 for GTMAC and CHPTAC, respectively).

### Crystallinity indices and lateral dimensions

The crystallinity indices (CrI) of unmodified cellulose and cationised derivatives were calculated from the areas of the crystalline and amorphous regions of C-4 using Equation 2 ( Newman, 1999).

$$Crl=\frac{Area\;of\;crystalline\;region\;of\;C-4}{Area\;of\;crystalline\;+amorphous\;regions\;of\;C-4}\;\times \;100\;(2)$$

Lateral dimensions ( *L*) were determined after spectral deconvolution using Equation 3 ( Newman, 1999) in which *h* is the thickness of a cellulose chain, determined to be 0.57nm ( Sugiyama *et al.,* 1991).

$$L=\frac{2h}{1-\sqrt{X}}\;(3)$$

*X* is the ratio of the crystalline fraction of C-4 i.e.

$$X=\frac{Area\;of\;crystalline\;region\;of\;C-4}{Area\;of\;crystalline\;+amorphous\;regions\;of\;C-4}\;(4)$$

### Adsorption experiments

The Cr(VI) removal efficiencies of cationised cellulose were determined by batch experiments at pH 4. To determine the optimal adsorbent mass for experiments, varying masses of CH-cellulose and GT-cellulose (3 – 120 mg) were added into 50 mL polypropylene tubes containing 20 mL Cr(VI) solutions (0.381 mg L ^-1^), and the tubes shaken on a horizontal rotary shaker at ambient temperature (21 ± 2 °C) for 24 hours. Adsorption kinetics were determined by monitoring Cr(VI) uptake from solutions at various time points (5 – 240 minutes), while isotherms were determined after exposing adsorbents to Cr(VI) solutions of concentrations ranging from 0.06 – 0.29 mg L ^-1^. At the end of reactions, adsorbents were separated from solutions by centrifugation and the supernatants filtered through 0.22 µm poly(vinylidene difluoride) (PVDF) syringe filters. The filtrates were then acidified using 5 % HNO _3_ and stored at 4 °C before analysis by inductively coupled plasma-mass spectroscopy (ICP-MS, Nexion 2000, Perkin Elmer, South Africa).

Cr(IV) equilibrium adsorption efficiencies of the adsorbents ( *q*_e_ (mg g ^−1^) were then calculated using Equation 5:

$$q_{e}=\frac{C_{o}-C_{e}}{m}\times V\;(5)$$

Where, *C*_0_ and *Ce* are the initial and final concentration of Cr(VI) in solution (mg L ^−1^), respectively, *V* is the volume of the solution (L) and *m* is the weight of the adsorbent (g).

### Application to real mine water

To determine the applicability of adsorbents to actual mine water samples, 0.1 g of each adsorbent was contacted with AMD-contaminated water (20 mL, pH 2.7), on a horizontal rotary shaker, for 4 hours (Previous experiments had shown this duration to be sufficient for equilibrium). After this time, they were centrifuged, filtered and acidified and stored at 4 °C before analysis. Cr(VI) concentrations in raw and treated water were determined by ICP-MS.

### Antibacterial activity assay

***Cell preparation.*** The antibacterial activities of the cationised adsorbents were determined by two approaches: cell viability and glutathione oxidation, using *Escherichia coli* as a model bacterium. *E. coli* cells were grown in Luria-Bertani broth at 37°C with constant agitation. Cells were harvested in the mid-exponential phase, centrifuged at 11,200 rpm for 1 minute and washed three times with sterile saline solution (0.9 % NaCl). Bacterial cell suspensions were then diluted to obtain cell samples containing 106 to 107 CFU mL ^-1^.

***Cell viability tests.*** Viability tests were conducted by incubating *E. coli* cells with unmodified and cationised cellulose (CH-cellulose and GT-cellulose) for 3 hours at room temperature (26°C). Suspensions were then used to create a series of dilutions that were plated and incubated at 37 °C overnight. Cells in isotonic saline solution but no cellulose materials was used as a control. The loss of viability of bacterial cells was determined by the colony counting method and calculated using the following equation:

$$Loss\;of\;viability=\frac{Counts\;of\;control-Counts\;of\;sample}{Counts\;of\;control}\;\times \;100\;(6)$$

***Glutathione oxidation tests.*** Strands of unmodified and cationised cellulose were placed into separate petri dishes containing 10 mL of 50 mM bicarbonate buffer (pH 8.6) and L-glutathione reduced (4 µL, 0.4 mM). The control contained the bicarbonate with the glutathione only. The petri dishes were then placed in a dark conditioned room at ambient temperature (26 °C) and shaken at 36 rpm for 3 hours. After this time, the suspension was filtered using a 0.45 µm poly(ether sulfone) syringe filters and the filtrate divided into four 15 mL Falcon tubes. Next, Tris-HCl buffer (1.57 mL, 1 M, pH 8.3) and Ellman’s reagent (30 µL, 100 mM) were added aliquots of the filtrate (900 µL) in 15 mL Falcon tubes, in duplicate. Glutathione loss was then determined using from absorbances recorded on a spectrophotometer at λ = 412 nm, using Equation 7:

$$\%\;Glutathione\;loss=\frac{Absorbance\;of\;Control-Absorbance\;of\;Sample}{Absorbance\;of\;Control}\;\times \;100\;(7)$$

## Results and discussion

### Characterisation

**Scheme 1.**
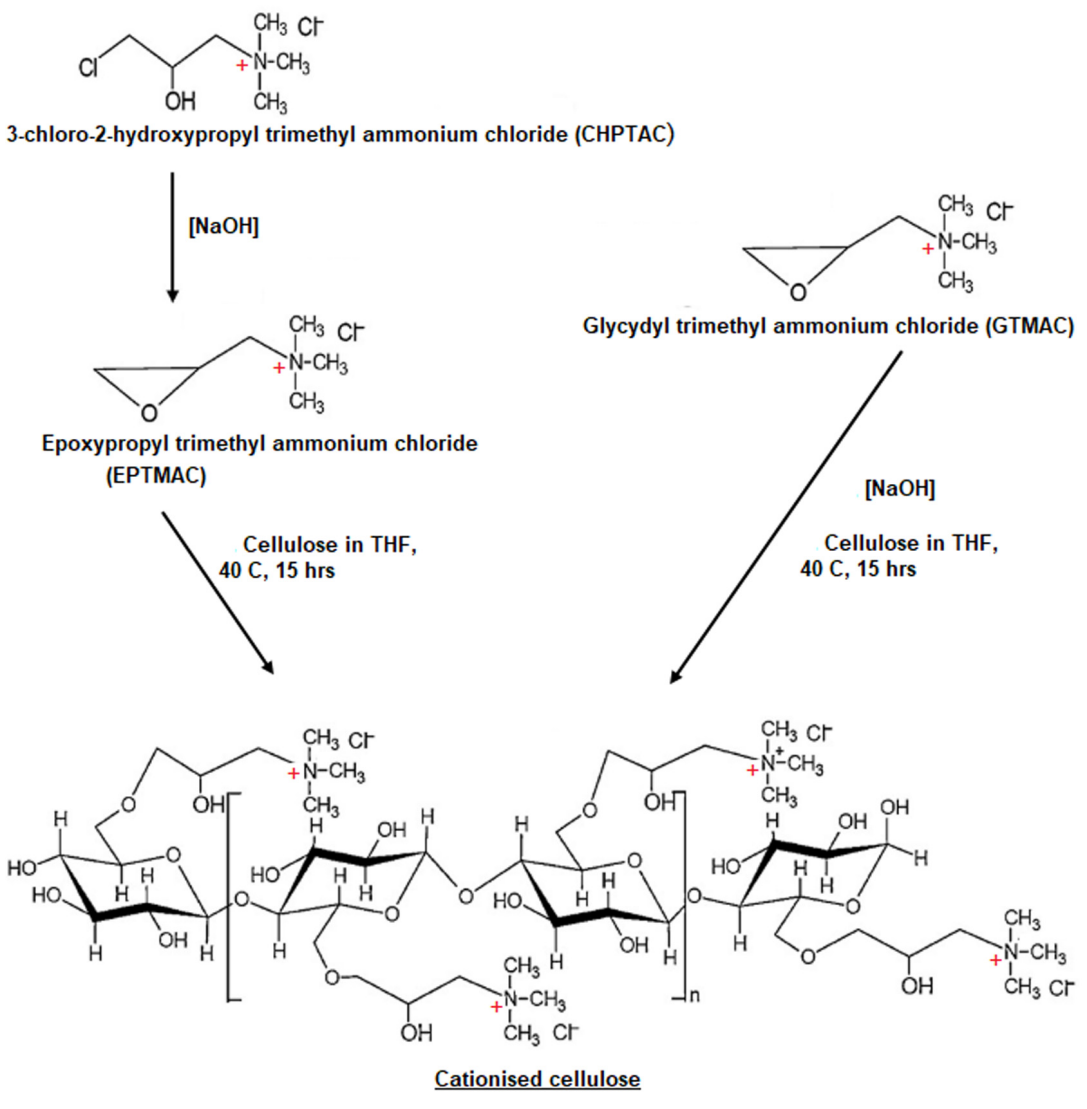
Schematic representation of cellulose cationization by CHPTAC and GTMAC.

In this work cellulose fibres extracted from hemp plants were cationised using CHPTAC and GTMAC, and NaOH as a catalyst as shown in Scheme 1. Cationization using CHPTAC proceeds in two steps: (a) the conversion of CHPTAC to 2,3-epoxypropyl trimethyl ammonium chloride (EPTMAC), which then reacts with cellulose fibres to generate cationised cellulose. Cationisation using GTMAC, on the other hand, proceeds in a more straightforward manner with no intermediate compounds. Nevertheless, both approaches involve reaction of the hydroxyl groups on the cellulose fibres with the epoxide ring of CHPTAC and GTMAC, leading to the grafting of quaternary ammonium salt moieties onto the cellulose fibres. Importantly, in both approaches, care must be taken to limit hydrolysis reactions as these reduces the efficiency of cationization. Because side reactions are promoted by the presence of water ( Zaman *et al.,* 2012), THF was used as a solvent. Odabas *et al.* (2016) showed that THF plays a “spectator” role, having little if any influence on the cationisation reaction.

**Figure 1.**
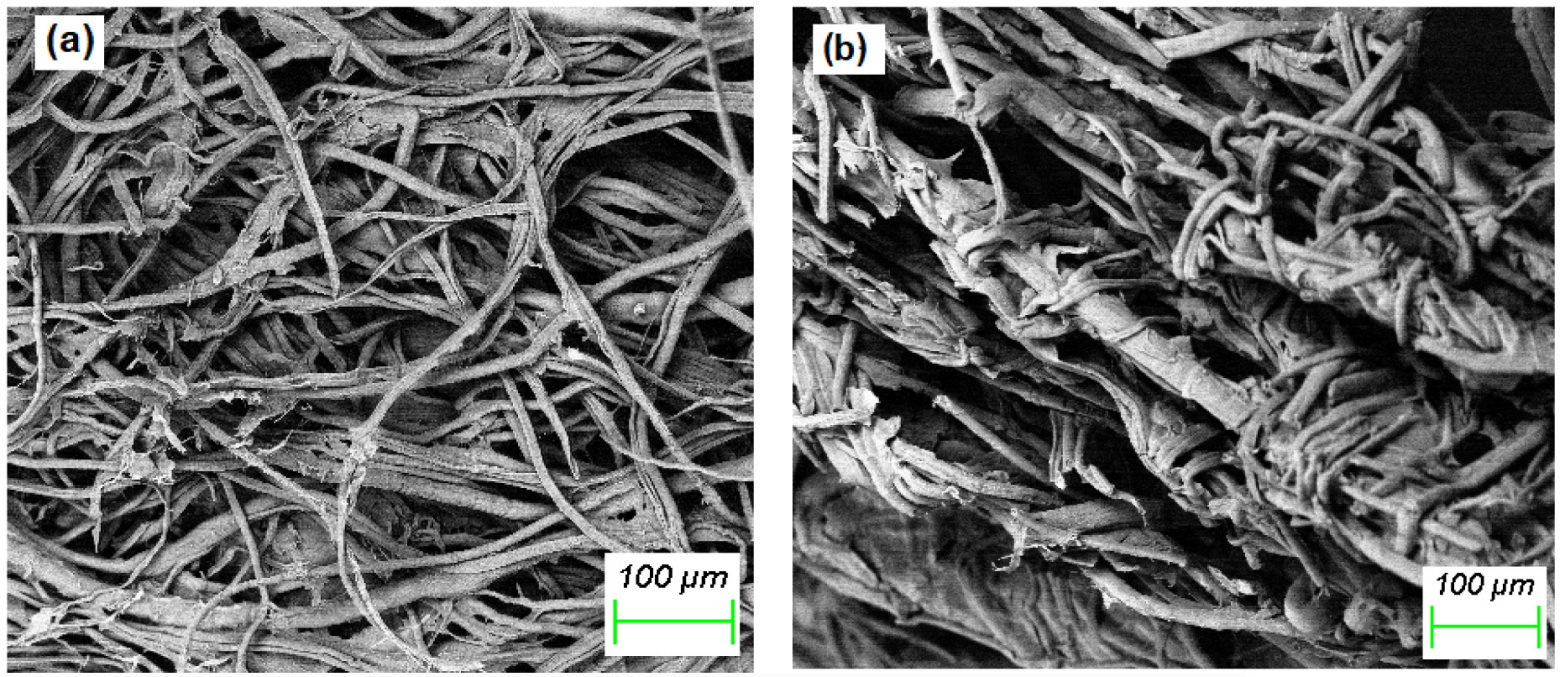
Scanning electron micrographs of ( **a**) CH-cellulose, and ( **b**) GT-cellulose.

Scanning electron micrographs ( Figure 1) show that both CH-cellulose and GT-cellulose adsorbents comprised assemblies of tangled fibres of varying dimensions. It was not possible, however, to determine fibril lengths as the fibril were entangled.

**Figure 2.**
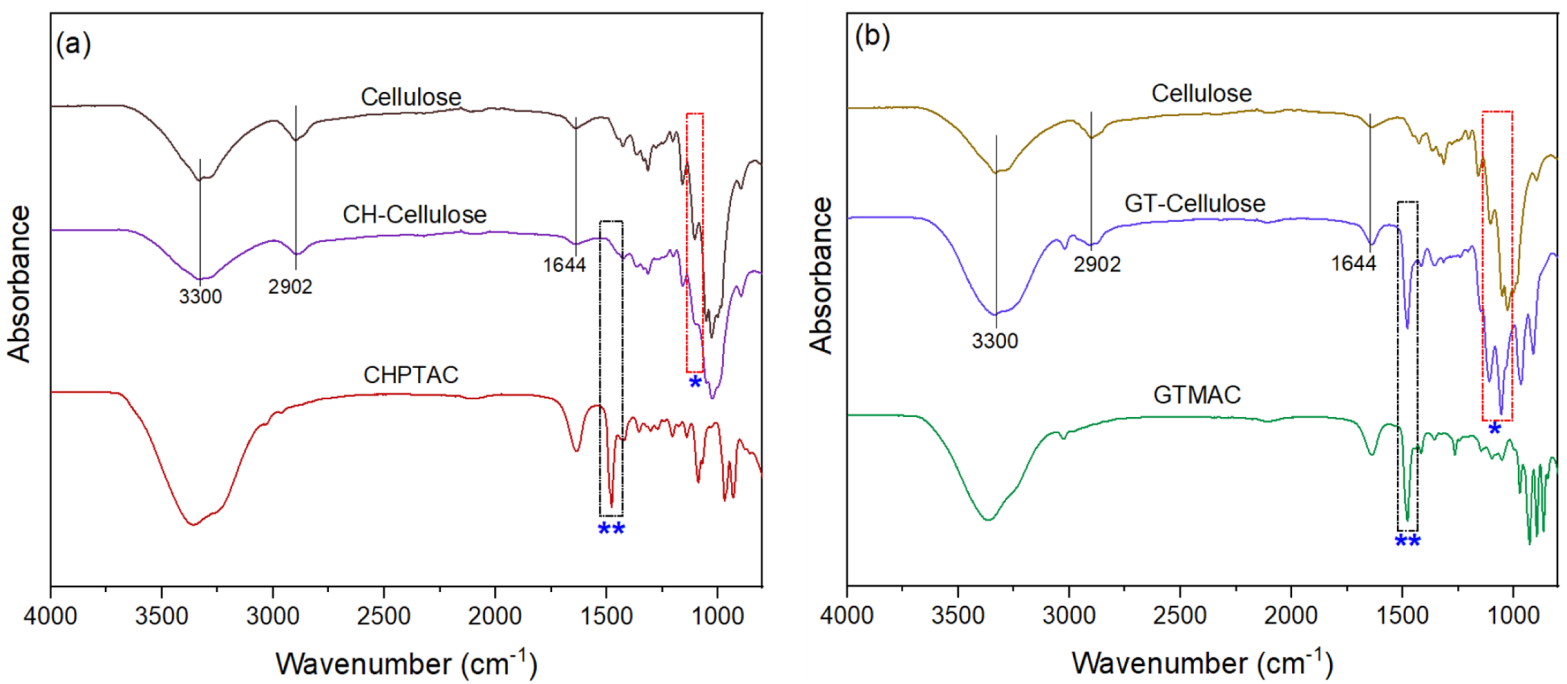
FTIR spectra of adsorbents before and after cationization with ( **a**) CHPTAC and ( **b**) GTMAC. Regions marked with a single asterisk (1056 cm ^-1 ^and 1100 cm ^-1^) represent ether linkages between cellulose C-6 and quaternary ammonium salts (see Scheme 1) while regions marked with two asterisks (1480 cm ^-1^) are due to vibrations of methyl groups of quaternary ammonium moieties.

FT-IR spectra of plain and cationised cellulose are shown in Figure 2. Characteristic absorption bands appeared in the spectra of both CH-Cellulose and GT-Cellulose at ~3300 and 2902 cm ^-1 ^ due to O-H and C-H moieties of cellulose, and at ~1644 cm ^-1 ^due to adsorbed water. However, the spectra of GTMAC-modified cellulose showed additional bands at 1056 and 1100 cm ^-1 ^ due to ether linkages formed between cellulose C-6 and the epoxy ring of the quaternary ammonium ion (see Scheme 1). A strong band at 1480 cm ^-1^ due to symmetric bending modes of the methyl groups of GTMAC was also observed ( Pour *et al.,* 2015; Zaman *et al.,* 2012). 

In contrast, an absorption band was not apparent at 1480 cm ^-1 ^in the spectra of CH-cellulose. Bands due to ether linkages were also weaker than those seen in GT-cellulose spectra suggesting that cationisation was much less with CHPTAC than with GTMAC. Indeed, calculations of the degree of substitution showed that the nitrogen content of CH-cellulose was ~21 times lower than that of GT-cellulose ( Table 1). We surmise that IR absorption of the ether bonds and methyl groups was below the limits of detection for FTIR. Nevertheless, elemental analysis confirmed the presence of amine groups in CH-cellulose.

**Table 1.** Elemental composition, degree of substitution, and pH _PZC_ values for unmodified cellulose and cationised derivatives.

	N (%)	C (%)	H (%)	S (%)	DS	pH *PZC*
Unmodified cellulose	0.03	45.89	7.53	0.01	-	5.6
CH-Cellulose	0.18	44.20	7.35	0.03	0.02	4.2
GT-Cellulose	3.85	52.29	10.70	0.05	0.50	7.4

**Figure 3.**
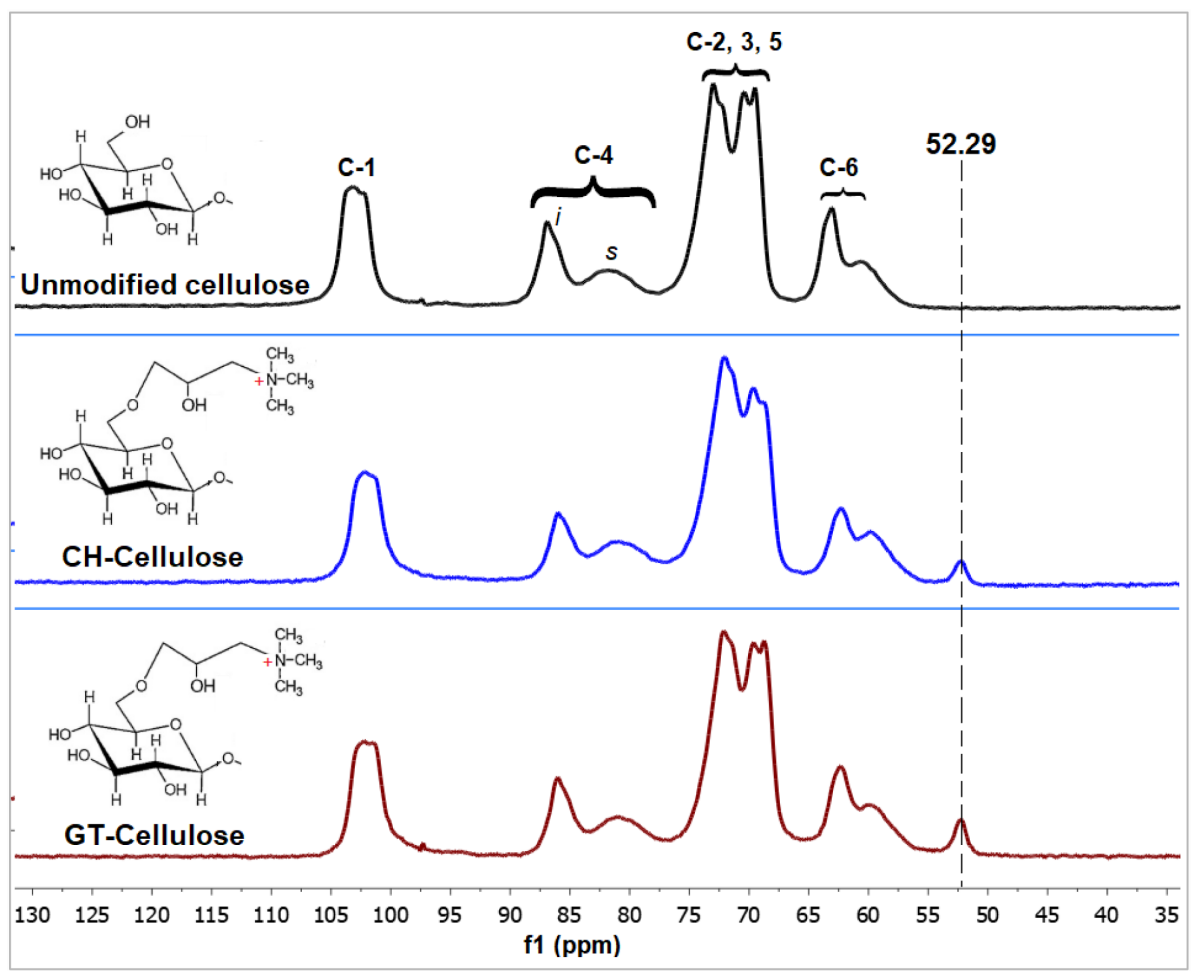
CP-MAS ^13^C NMR Spectra of unmodified and cationised cellulose, showing crystalline ( *i*) and amorphous ( *s*) regions of C-4.

The CP-MAS ^13^C NMR spectra of cellulose extracted from hemp fibres, and cationised derivatives are presented in Figure 3. Unmodified cellulose had spectra typical of cellulose I allomorph ( Table 2) ( Newman, 1999; Wickholm *et al.,* 1998). The resonances at 102–103 ppm were assigned to C-1 carbons while those at 80–86 ppm were from C-4 glycosidic bond carbons. The resonances observed at 68 – 73 ppm are from C-2, C-3, and C-5 of the pyranoid ring while those at 59-63 ppm are from C-6 carbons. Cationised celluloses had a new peak at ~52 ppm from (CH _3_) _3_N ^+^- groups of the quaternary amines, confirming the incorporation of CHPTAC and GTMAC in the cellulose structure as shown in Scheme 1.

**Table 2.** ^13^C CP-MAS NMR chemical shift assignments (in ppm), crystallinity indexes ( *CI*), and lateral dimensions ( *L*) of cellulose and cationised derivatives.

Sample	Chemical shift (ppm)									
	C-1		C-2, 3, 5		C-4		C-6		*CI (%)*	*L* (nm)
Cellulose	103.57	102.26	73.16	69.42	86.96	81.77	63.21	60.28	50	3.88
CH-Cellulose	102.76	101.39	72.10	68.57	85.86	81.01	62.40	59.58	40	3.08
GT-Cellulose	102.80	101.41	72.07	68.58	85.90	80.99	62.41	59.47	44	3.42

With respect to crystallinity of cellulose I, the C-4 region of the NMR spectrum is the most informative ( Wickholm *et al.,* 1998). The broad peak represents the amorphous region while the sharp peak represents the crystalline region. Cationised derivatives had lower crystallinity indexes compared to the parent cellulose ( Table 2). This was due to a decrease in the ratio of crystalline to amorphous regions from ~1 in the parent cellulose to 0.8 in GT-cellulose and 0.7 in CH-cellulose. The lateral dimensions also followed a similar trend, decreasing from 3.88 nm in unmodified cellulose, to 3.08 nm (CH-cellulose). Cationisation therefore decreased fibril diameter. We posit that reaction with NaOH during cationisation broke down the amorphous regions of cellulose fibres further, reducing fibril dimensions. Similar findings were reported by Heux and colleagues for sugar beet cellulose ( Heux *et al.,* 1999). NMR output data are available (see *Underlying data*, Etale *et al.* (2020a)).

### Cr(VI) adsorption performance of cationised cellulose

**Figure 4.**
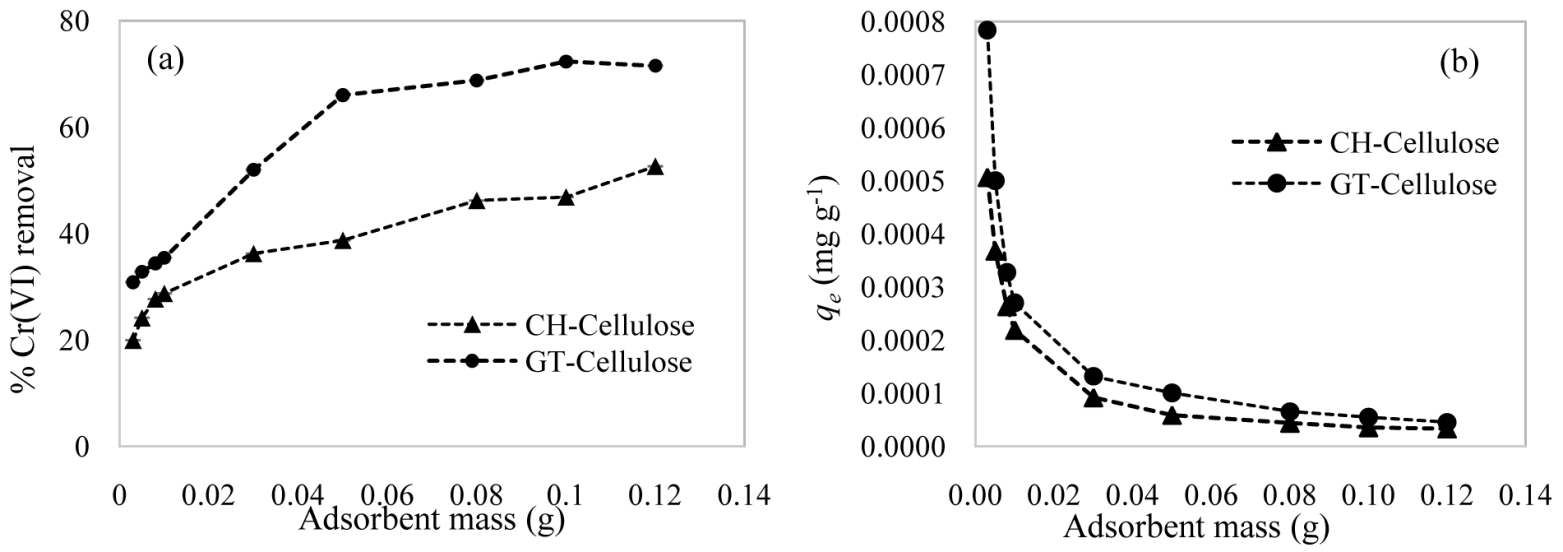
Effect of adsorbent dose on ( **a**) % Cr(VI) removal by CH-cellulose and GT-cellulose. ( **b**) Experimental *q _e_* values. Conditions: pH = 4, [Cr(VI)] = 0.381 mg L ^-1 ^, contact time = 240 minutes.

The percentage removal of Cr(VI) by cationised cellulose at pH 4 is presented in Figure 4. For these experiments, varying amounts of CH-cellulose and GT-cellulose were exposed to Cr(VI) solutions (0.381 mg L ^-1^) at pH 4 for 240 minutes. The results showed that Cr(VI) uptake increased with increasing adsorbent concentration and that it was higher for GT-cellulose than CH-cellulose. The increase in Cr(VI) removal with increasing adsorbent dosage is due to the increase in available binding sites on the adsorbent. Importantly, though, these data suggest that GT-cellulose was a more efficient Cr(VI) adsorbent than CH-cellulose. This may be explained by differences between the two materials with respect to (i) availability of protonated amine sites based on nitrogen concentration, and (ii) the pH _PZC_ of the adsorbents ( Table 1). GT-cellulose had a higher concentration of quaternary amines in the matrix, as evidenced by the higher concentration of nitrogen. Further, with a pH _PZC _of 7.4, most of these amine sites were protonated at the experimental pH of 4. In contrast, most sorption sites of CH-cellulose fibres are neutral at pH 4 due to the pH _PZC _of 4.2 of this material. As such, electrostatic attraction between CH-cellulose and HCrO _4_^-^ ions were limited, hence the low adsorption efficiency. As the adsorption efficiency of GT-cellulose became constant above 0.1 g of adsorbent mass, this was set as the adsorbent concentration for subsequent studies for both materials in order to generate comparable data. 

### Adsorption kinetics

The rate of adsorption is one of the most important aspects defining the efficiency of an adsorbent. In this study, the effect of time on the uptake of Cr(VI) ions by cationised celluloses was investigated at pH 4, using 0.1 g of adsorbents and 20 mL of adsorbate (0.381 mg L ^-1^). Adsorption data were then subjected to the pseudo-first-order, pseudo-second-order, and Elovich kinetics models represented by Equation 8, Equation 9 and Equation 10, respectively ( Ho & Mckay, 1998).

$$\ln(q_{e}-q_{t})=\ln(q_{e})-k_{1}\;t\;(8)$$

$$\frac{t}{q_{t}}=\frac{1}{k_{2}q_{e}^{2}}+\frac{1}{q_{e}}t\;(9)$$

$$q_{t}=\beta \;\ln(\alpha \beta )+\ln\;(t)\;(10)$$

Model fit and rate constants were determined from plots of ln( *q _e_ - q _t_*) versus *t* for the pseudo-first order model, *t/q _t_* against *t* for the pseudo-second order model, and *q _t_* against ln( *t*) for the Elovich model. The results show that the rate of Cr(VI) uptake by cationised cellulose was best fitted by the pseudo-second order model ( Table 3), suggesting that chemical sorption was the rate-limiting step of the process. Data used to assess adsorption kinetics are available (see *Underlying data*, Etale *et al.* (2020a)).

**Table 3.** Kinetics model parameters for the uptake of Cr(VI) onto cationised cellulose. Conditions: pH = 4, [Cr(VI)] = 0.381 mg L ^-1 ^, adsorbent mass = 0.1g.

Model	Parameter	CH-Cellulose	GT-Cellulose
Pseudo-first-order	*k*_1_ (min ^-1^)	0.0002	0.0007
	*q*_e, cal_ (mg g ^-1^)	0.0003	0.0002
	*q*_e, exp_ (mg g ^-1^)	0.0008	0.0013
	*R* ^2^	0.3680	0.2065
Pseudo-second-order	*k*_2_ (g mg ^-1^ min ^-1^)	285.96	780.16
	*q*_e, cal_ (mg g ^-1^)	0.0008	0.0013
	*q*_e, exp_ (mg g ^-1^)	0.0008	0.0013
	*R* ^2^	0.9993	0.9996
Elovich	*α* (g mg ^-1^ min ^-1^)	0.0779	0.2537
	*ꞵ* 1/b (g mg ^-1^)	15283.58	9658.08
	*R* ^2^	0.8636	0.5653

### Adsorption isotherms

Adsorption isotherms explain adsorbate-adsorbent interactions at sorption sites. This information helps to understand sorption mechanisms, as well as for the design of appropriate sorption systems. In order to understand the mechanisms by which Cr(VI) ions were sorbed to cationised cellulose, experimental sorption data subjected to three isotherm models: Langmuir, Freundlich, and Dubinin–Radushkevich.

The Langmuir isotherm is based on a number of assumptions including a fixed number of sorption sites on an adsorbent surface and a lack of dependence on surface coverage, of the free energy of adsorption. As such, adsorption of an ion to a particular site is independent of whether adjacent sites are occupied by solvent or other adsorbate species. In other words, the driving force for sorption of an ion to an adsorbate-free site is similar to that for adsorption to a nearly filled surface. The assumption of a fixed number of sorption sites means that as more ions sorb to the adsorbent surface and sorption sites decrease, and the tendency of sorption of additional sorbate molecules decreases. Nevertheless, whenever a sorbate molecule sorbs to a site, the free energy is the same as that involved in the formation of previous adsorbate-sorbent bonds ( Leckie *et al.,* 1980).

It is represented by Equation 11 below where *q _m_* is the maximum adsorption capacity (mg g ^-1^) and *K _L_* is the Langmuir constant which is related to adsorption energy (L mg ^-1^). *K _L_* and *q _m_* are determined from a plot of 1/ *q _e_* versus 1/ *C _e_*.

$$\frac{1}{q_{e}}=\left(\frac{1}{q_{m}}\right)\;+\frac{1}{K_{L}q_{m}\;C_{e}}\;(11)$$

The Freundlich isotherm ( Equation 12) assumes that sorbent sites are heterogeneous in terms of their energies, and that ions can form more than single layer on the sorbent surface. In a qualitative way, *K*_F_ is related to the strength of the sorption bond, and *n* to the distribution of bond strengths ( Leckie *et al.,* 1980). The greater the difference between the *n* value of a reaction and 1, the wider is the distribution of surface bond energies of sorbate-sorbent interaction for that adsorbent i.e. greater heterogeneity of sorption sites.

$$\log q_{e}=\left(\frac{1}{n}\right)\log C_{e}+\log K_{F}\;(12)$$

An *n* < 1, suggests bond energies increasing with adsorption density, which may occur, when interactions between ions sorbed onto of the adsorbent are favourable. Conversely, *n* > 1 suggests unfavourable adsorbate-adsorbate interactions.

The Dubinin–Radushkevich (D-R) model also assumes multi-layer adsorption, but proceeds from the premise that sorbate molecules bind first to energetically favourable sites, before multilayer adsorption can begin ( Hutson & Yang, 1997). It is described by Equation 13 in which *K*_D-R_, (mol ^2^ J ^-2^) is a constant related to the adsorption energy, and ε is the Polanyi potential. The latter is calculated using Equation (14) in which *R* is the gas law constant (kJ mol ^-1^ K ^-1^) and *T* is the absolute temperature (K). *K*_D-R_ and *q _m_* can be determined from a plot of ln *q _e_* versus *ε.*

$$lnq_{e}=lnq_{m}–K_{D-R}\;\varepsilon^{2}\;(13)$$

$$\varepsilon =RTln\;\;\left(1+\frac{1}{C_{e}}\right)\;(14)$$

The D-R isotherm also allows for evaluation of the nature of adsorption i.e. by chemisorption or physisorption, based on an evaluation of the mean energy of adsorption ( *E*, kJ mol ^-1^) that can be determined using Equation 15.

$$E=\frac{1}{\sqrt{2}K_{D-R}}\;(15)$$

A value of *E* < 8 kJ mol ^-1^ represents physisorption while a value between 8 and 16 kJ mol ^-1 ^is representative of chemisorption. When E is >16 kJ mol ^-1^, adsorption is likely dominated by particle diffusion.

Results of model fitting of adsorption data to the three isotherms described above are given in Table 4. Based on the *R ^2^* values, Cr(VI) sorption to both cationised cellulose derivatives was best described by the Freundlich and D-R models ( *R ^2^* > 0.95 *)*. This suggests that adsorption of Cr(VI) ions to the surfaces of these sorbents involves binding to energetically heterogeneous sites, and in multiple layers.

**Table 4.** Parameters of isotherms for Cr(VI) adsorption by cellulose modified by CHPTAC and GTMAC.

Isotherm	Parameter	CH-Cellulose	GT-Cellulose
Dubinin–Radushkevich (D-R)	*K*_D-R_, (mol ^2^ J ^-2^)	0.007	0.005
	*q _m_*_(cal)_ (mmol g ^-1^)	0.983	1.010
	*E*, kJ mol ^-1^	8.260	9.827
	*R ^2^*	0.970	0.953
Freundlich	*K*_F_ ((mg g ^-1^) (mg L ^-1^) ^1/n^)	1.010	1.012
	*n*	1.633	2.030
	1 */n*	0.612	0.493
	*R ^2^*	0.975	0.951
Langmuir	*R ^2^*	0.006 ^*^	0.507 ^*^

*Parameters not calculated due to the low *R ^2^* value.

Furthermore, as the value of *E* from the D-R isotherm, was between 8 and 16 kJ mol ^-1^ in both cases, this suggests that the binding of Cr(VI) to surfaces of cationic cellulose involved chemisorption. From the Freundlich isotherm, *n* was found to be >1 for both materials, implying that interactions between sorbed Cr(VI) on the adsorbent surface were not favourable for adsorption e.g. due to repulsive forces between negatively charged ions. As a result of the anion-anion repulsions, approach of additional anions towards the adsorbate surface was hindered, thus limiting adsorption efficiency of the materials ( Hu *et al.,* 2005; Leckie *et al.,* 1980). Data used to assess adsorption isotherms are available (see *Underlying data*, Etale *et al.* (2020a)).

### Application to AMD- contaminated water

**Figure 5.**
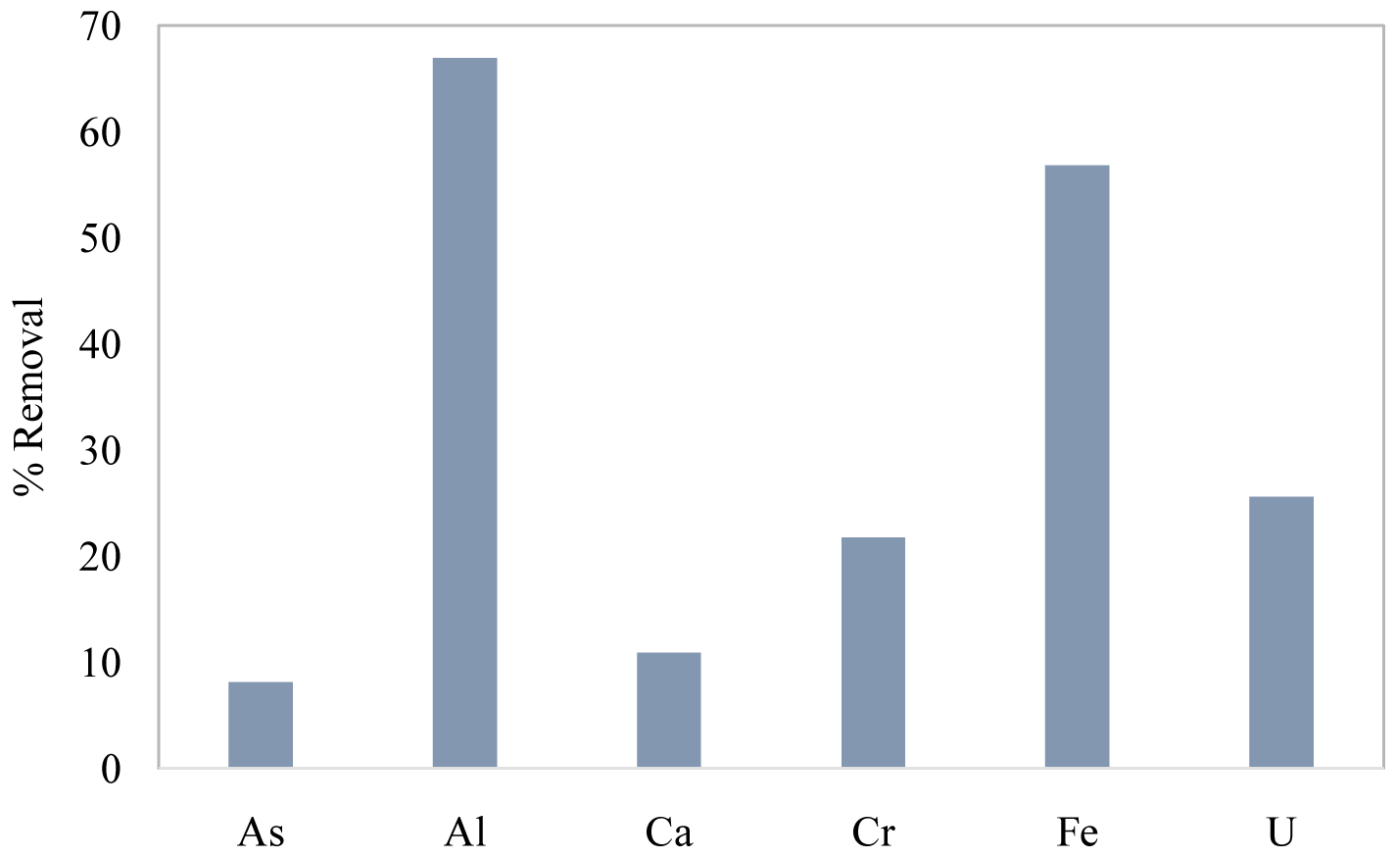
Removal of Cr(VI) and other ions present in mine drainage contaminated water by GT-cellulose. *Conditions:* pH = 2.7, adsorbent mass = 0.1 g, Volume = 20 mL, contact time = 240 minutes.

As GT-cellulose showed greater efficiency for Cr(VI) removal in synthetic water samples, it was selected to study uptake of Cr(VI) from AMD-contaminated water. The results of experiments conducted for 240 minutes without any pH adjustments to the water are presented in Figure 5 below. These data show that Cr(VI) removal by GT-cellulose decreased from 72 % in single-ion solutions, to 22 % in AMD contaminated water. The data show that this is likely due to competition effects from other ions present in AMD, particularly Al and Fe. We surmise that because these ions exist at much higher concentrations, they deposit onto cellulose fibres, blocking Cr(VI) sorption sites on GT-cellulose. This implies that the effective use of cationic cellulose for Cr(VI) removal would require pre-treatment steps involving removal of Fe and Al.

### Antibacterial activity of cationised cellulose

The use of cationisation agents as sorbents for water treatment proffers additional benefits beyond the removal of anions due to antimicrobial properties of quaternary ammonium salts ( Jennings *et al.,* 2015; Pour *et al.,* 2015). The antimicrobial properties of cationised adsorbents prepared in this study were quantified using *E. coli* as a model indicator bacterial species. Two techniques were employed: cell viability and glutathione loss. The loss of viability by bacterial cells in the presence of quaternary ammonium salts begins when the positively charged heads of the salts bind electrostatically to the phospholipid membranes of bacteria. This results in membranes being disrupted or thinned, leakage of cytosolic contents, and destruction of essential gradients, culminating in the death of cells ( Jennings *et al.,* 2015).

Cell death may also be via reactive oxidative species (ROS) ( Liu *et al.,* 2011). The glutathione test offers an indirect way of measuring ROS production by bacterial cells. Glutathione is present in bacteria as an antioxidant. In the presence of ROS, its thiol groups are oxidised to disulphide bonds. In the glutathione test, Ellman’s test is used to test for ROS production by quantifying thiol concentrations. Reduction in glutathione concentration indicates ROS production.

The results of cell viability tests and glutathione oxidation are presented in Table 5. They show that exposure to cationised cellulose reduced the viability of *E. coli* from ~72% (unmodified cellulose) to ~56% (GT-cellulose). This confirms the known effects of quaternary ammonium salts and suggests that prepared adsorbents could proffer disinfection advantages. Further, the greater antibacterial activity GT-cellulose implies that antimicrobial efficiency of the adsorbents can be increased by optimisation of cationisation.

**Table 5.** Loss of viability for *E. col* *i*, and glutathione oxidation induced by unmodified cellulose and cationised derivatives.

	Loss of Viability of *E. coli* cells (%)	Loss of Glutathione (%)
Control	0	0
Unmodified cellulose	27.7	30.4
CH-cellulose	34.9	30.5
GT-cellulose	44.5	36.9

To further explore the possibility that ROS played a role in cell death, glutathione experiments were conducted. The loss of glutathione, particularly for GT-cellulose, suggests that the presence of cationised cellulose resulted in production of ROS. This implies that cationised cellulose mediated oxidative stress in *E. coli*. We posit that the loss of cell viability may be due, in part, to oxidation of thiols. Furthermore, this oxidation may be of phospholipids thus compromising membrane structure, but it may also be of cellular components including proteins, lipids and DNA. Results of glutathione experiments and antibacterial cell counts are available (see *Underlying data*, Etale *et al.* (2020a)).

## Conclusion

Cellulose cationised using CHPTAC and GTMAC were found to be effective sorbents for the removal of Cr(VI) ions from synthetic and actual samples of mine contaminated water. Cellulose cationised using GTMAC showed greater removal capacities at pH 4, likely due to its higher cationisation efficiency as determined by elemental analysis. The pH _PZC_ of materials also influenced Cr(VI) removal. With a pH _PZC_ of 4.2, the surfaces of CHPTAC-cationised cellulose were almost all neutrally charged at pH 4 resulting in negligible electrostatic attraction between adsorbent sites and the adsorbate ions. In contrast, GTMAC-cationised cellulose with a pHPZC of 7.4, and was positively charged at the experimental pH and primed for anion uptake. For both adsorbents, reaction kinetics followed the pseudo-second order model, while isotherms were best described by the Freundlich and Dubinin-Radushkevich models. The adsorbents were also found to have significant antimicrobial properties, inducing the production of reactive oxidative species and reducing the viability of *E. coli* cells from ~77% to ~56%. Together, these results suggest that cellulose cationisation can be a useful approach for the development of environmentally-friendly and affordable adsorbents for the removal of Cr(VI) ions from mine-drainage contaminated water. Further, these adsorbents would offer the additional advantage of disinfection that could be useful where the water is also subjected to biological contamination. Nevertheless, pre-treatments to reduce Al and Fe loads would be required in order to increase the adsorbent efficiencies.

## Data availability

### Underlying data

Figshare: Synthesis and application of cationised cellulose for removal of Cr(VI) from acid mine-drainage contaminated water. https://doi.org/10.6084/m9.figshare.13299128.v2 ( Etale *et al.,* 2020a).

This project contains the following underlying data:

- Data File 1-SEM Images.docx
- Data File 2-CHNS data.xlsx. (Results of elemental analyses of unmodified and modified cellulose.)
- Data File 3.1-Plain Cellulose NMR.zip
- Data File 3.2-GTMAC-Cellulose NMR.zip
- Data File 3.3-CHPTAC-Cellulose NMR.zip
- Data File 4.1-Adsorption Kinetics.xlsx. (Data for Cr(VI) adsorption kinetics.)
- Data File 4.2-Adsorption isotherms.xlsx. (Data for Cr(VI) adsorption isotherms.)
- Data File 4.3-Effect of adsorbent mass.xlsx (Data for Cr(VI) adsorption mass.)
- Data File 5-Antibacterial tests.xlsx. (Cell viability counts and glutathione oxidation results.)
