# Supplementary material for: Synthesis and application of cationised cellulose for removal of Cr(VI) from acid mine-drainage contaminated water
Source: AAS Open Res. 2021 Jan 21;4:4. [Version 1] doi: 10.12688/aasopenres.13182.1 (PMC7871422; doi:10.12688/aasopenres.13182.1)
Supplement: Peer review history

# Peer review: Reviewer response for version 1

**DOI:** 10.21956/aasopenres.14289.r28296

**Published:** 2021-02-09

**Authors:** Balla Diop Ngom

**Affiliations:** 1 Laboratory of Quantum Photonics, Energy and Nano-Manufacturing, Faculty of Sciences and Techniques, Cheikh Anta Diop University of Dakar (UCAD), Dakar, Senegal

The authors have presented an interesting work, the way that the work has been conducted is well described and the manuscript is well written and the language is well defined.

1. However the authors are claiming to develop and green and sustainable approach for the production of plant based biomass derived nanocellulose. The authors should explain why the use of chemicals since they are claiming developing an green approach.
2. The nanocellulose production has goes through a first step of alkali treatment using 4 wt% NaOH at 80 °C in three treatment rounds, each lasting 2 hours. The authors should give more details how these figures have been chosen.
3. The authors claimed that "The results of cell viability tests and glutathione oxidation are presented in Table 5. They show that exposure to cationised cellulose reduced the viability of *E. coli* from ~72% (unmodified cellulose) to ~56% (GT-cellulose). This confirms the known effects of quaternary ammonium salts and suggests that prepared adsorbents could proffer disinfection advantages. Further, the greater antibacterial activity GT-cellulose implies that antimicrobial efficiency of the adsorbents can be increased by optimization of cationization" how these results are compared to the data published into the literature.

Is the work clearly and accurately presented and does it cite the current literature?

Yes

If applicable, is the statistical analysis and its interpretation appropriate?

Not applicable

Are all the source data underlying the results available to ensure full reproducibility?

Yes

Is the study design appropriate and is the work technically sound?

Yes

Are the conclusions drawn adequately supported by the results?

Partly

Are sufficient details of methods and analysis provided to allow replication by others?

Yes

Reviewer Expertise:

Materials Science and Engineering

I confirm that I have read this submission and believe that I have an appropriate level of expertise to confirm that it is of an acceptable scientific standard.

# Peer review: Reviewer response for version 1

**DOI:** 10.21956/aasopenres.14289.r28293

**Published:** 2021-02-08

**Authors:** Gift Mehlana

**Affiliations:** 1 Department of Chemical Technology, Faculty of Science and Technology, Midlands State University, Gweru, Zimbabwe

This study reports the synthesis and use of cationised cellulose for removal of chromium from waste water. The work is well written and the general presentation is good. Appropriate techniques were used to characterise the prepared materials. The conclusions drawn in this study are scientifically correct. I recommend this work being indexed.

Is the work clearly and accurately presented and does it cite the current literature?

Yes

If applicable, is the statistical analysis and its interpretation appropriate?

Yes

Are all the source data underlying the results available to ensure full reproducibility?

Yes

Is the study design appropriate and is the work technically sound?

Yes

Are the conclusions drawn adequately supported by the results?

Yes

Are sufficient details of methods and analysis provided to allow replication by others?

Yes

Reviewer Expertise:

Materials chemistry

I confirm that I have read this submission and believe that I have an appropriate level of expertise to confirm that it is of an acceptable scientific standard.

# Peer review: Reviewer response for version 1

**DOI:** 10.21956/aasopenres.14289.r28298

**Published:** 2021-02-08

**Authors:** Cecil K. King'ondu

**Affiliations:** 1 Department of Chemical and Forensic Sciences, Botswana International University of Science and Technology, Palapye, Botswana; 2 Department of Physical Sciences, South Eastern Kenya University, Kitui County, Kenya

**General comment**

 The work presented herein on synthesis and surface functionalization of cellulose and application of functionalized cellulose in removal of Cr(VI) ions is good for AAS readership and represents a significant contribution to the field(s) of water quality and wastewater treatment. I therefore recommend it for indexing subject to addressing the following minor comments.

**Specific comments**

**Abstract:**

 Consider revising the sentence reading..."Nevertheless, 47%-72% of Cr(VI) ions were removed from solutions at pH 4, BY 0.1 g of  CHPTAC and GTMAC-cationised cellulose, respectively"' ... to read ........ *""Nevertheless, 47 and 72% of Cr(VI) ions were removed from solutions at pH 4, BY 0.1 g of  CHPTAC and GTMAC-cationised cellulose, respectively"'*

**Results and Discussion**

 It would be good for the readers of this work to know how pristine/unfunctionalized cellulose compares with CH- and GT-cellulose in terms of removing Cr(VI) ions. Therefore, the authors should include the performance of pristine cellulose in Figure 4a,b.

 Consider revising the sentence reading...."at 3300 amd 2902 cm ^-1^ due to O-H and C-H moieties of cellulose"'.... to read...."""at 3300 amd 2902 cm ^-1^ due to O-H and C-H moieties of cellulose, respectively"'

 In scheme 1, the top-right nitrogen of cationised cellulose has two positive charges. Consider deleting one.

 The authors attribute the difference in the performance of the two materials CH- and GT-cellulose to their difference in terms of available of protonated amine sites and pH _PZC_. However, there are no amine sites in both cationised cellulose materials to be protonated but quaternary ammonium sites which cannot be protonated. From the FTIR spectra (Fig2) it is clear that CH-cellulose had little to no ether linkages as evinced by weak bands at 1056 and 1100 cm-1 compared to those of its GT-cellulose counterpart which further suggest less cationization in CH-cellulose.

 Therefore the authors should consider attributing  the superior performance of GT-cellulose to protonation of ether groups not amine sites.

Is the work clearly and accurately presented and does it cite the current literature?

Yes

If applicable, is the statistical analysis and its interpretation appropriate?

Yes

Are all the source data underlying the results available to ensure full reproducibility?

Yes

Is the study design appropriate and is the work technically sound?

Yes

Are the conclusions drawn adequately supported by the results?

Yes

Are sufficient details of methods and analysis provided to allow replication by others?

Yes

Reviewer Expertise:

Nanomaterials for catalysis, wastewater treatment, energy storage among others

I confirm that I have read this submission and believe that I have an appropriate level of expertise to confirm that it is of an acceptable scientific standard.

# Peer review: Reviewer response for version 1

**DOI:** 10.21956/aasopenres.14289.r28295

**Published:** 2021-01-28

**Authors:** Andrew C. Eloka-Eboka

**Affiliations:** 1 School of Chemical and Minerals Engineering, North West University, Potchefstroom, South Africa; 2 AAS, Potchefstroom, South Africa

The study evaluated the synthesis and application of cationised cellulose from hemp for the removal of Cr ions from acid mine drainage contaminated water and evaluation of the the efficacy of Cr ion removal thereof. The study is worthy of investigation looking at the adverse implication of toxic metals like chromium in drinking water. The approach and methodology were appropriate and adequate and the results/implication of study projected that the cationised cellulose were effective sorbents even at a pH of 4. There are also antibacterial potentials of the sorbents. Overall cationisation of adsorbents offers useful advantage in disinfecting water from biological contamination.

Is the work clearly and accurately presented and does it cite the current literature?

Yes

If applicable, is the statistical analysis and its interpretation appropriate?

Yes

Are all the source data underlying the results available to ensure full reproducibility?

Yes

Is the study design appropriate and is the work technically sound?

Yes

Are the conclusions drawn adequately supported by the results?

Yes

Are sufficient details of methods and analysis provided to allow replication by others?

Yes

Reviewer Expertise:

Water and wastewater treatment, bio-processng, bio-energy, environment

I confirm that I have read this submission and believe that I have an appropriate level of expertise to confirm that it is of an acceptable scientific standard.
